# Predisposition and Working Conditions for the Occurrence of Lumbar Syndrome in Medical Workers of the Clinical Center of Montenegro during the COVID-19 Pandemic

**DOI:** 10.3390/jcm13082431

**Published:** 2024-04-21

**Authors:** Dragana Jovanović, Dragana Backović, Ana Tomas, Zoran Bukumirić, Bojan Koprivica

**Affiliations:** 1 Clinical Center of Montenegro, 81000 Podgorica, Montenegro; dragana.j@ucg.ac.me (D.J.); b.dragana@ucg.ac.me (D.B.);; 2 Faculty of Medicine, University of Montenegro, 81000 Podgorica, Montenegro; 3 Department of Pharmacology and Toxicology, Faculty of Medicine Novi Sad, University of Novi Sad, 21000 Novi Sad, Serbia; 4 Institute of Medical Statistics and Informatics, Faculty of Medicine, University of Belgrade, 11080 Belgrade, Serbia; zoran.bukumiric@med.bg.ac.rs

**Keywords:** lumbar syndrome, COVID-19, medical workers, Montenegro

## Abstract

**Background:** Lumbar pain is a condition of discomfort in the lower back caused by numerous factors, lasting for short or longer periods of time. Healthcare professionals, regardless of the type of care they are engaged in, are at risk of lumbar pain. This is the first study that deals with the problem of lumbar syndrome in health workers in Montenegro. **Methods:** This cross-sectional study included full-time health workers employed in the Clinical Center of Montenegro who were involved in the treatment of COVID-19 patients during 2020 and 2021. The survey consisted of general questions for collecting socio-demographic and COVID-19 engagement data; the Modified Nordic questionnaire was used for the analysis of musculoskeletal symptoms, and the EQ-5D—questionnaire was used to measure the quality of life associated with health. **Results:** The one-year prevalence of lumbar pain was 68.1%. Factors associated with lumbar pain were as follows: a higher degree of physical inactivity (each subject with a higher degree of physical inactivity had a 24% higher chance of occurrence of lumbar pain); a higher degree of load and over-engagement during the COVID-19 pandemic (each subject with a higher degree of workload had a nearly 50% higher chance of occurrence of lumbar pain); duration of engagement during the COVID-19 pandemic (subjects engaged up to a month were 4 times more likely to develop lumbar pain, and subjects engaged for 1–3 months were 3.5 times more likely to develop lumbar pain compared to those who were not engaged in COVID-19 treatment). This study also confirms that lumbar syndrome affects the quality of life of health workers. **Conclusions:** Lumbar syndrome is highly prevalent among healthcare professionals in the Clinical Center of Montenegro, especially in the population of nurses, where evidence-based preventive measures are needed.

## 1. Introduction

Lumbar pain is a condition of discomfort in the lower back, between the 12th rib and the gluteus fold, which can be caused by various factors and last for a shorter or longer period of time. Lower back pain is among the leading causes of disability, occurring at similar rates in all cultures and interfering with quality of life and performance at work [1]. Few cases of back pain are due to specific causes, and the pain mostly presents as acute and usually as self-limiting [1]. The occurrence of low back pain causes difficulty in actively controlling the movement of the lower back, which interferes with daily activities. It has been identified as one of the most common musculoskeletal disorders. Lumbar pain is considered to be one of the most common health problems nowadays and, at the same time, is identified as the most common reason for temporary work incapacity [2].

In healthcare professionals, the risk of lumbar pain exists in all employees regardless of the type of healthcare activities they are engaged in. Personal (history of back problems) and ergonomic factors (more frequent patient handling) are reported among the variables contributing to new episodes of lower back problems [3,4]. In addition to these, organisational factors, decreasing staffing levels, and increasing working hours may exacerbate the issue. Healthcare professionals are exposed to a variety of occupational risks, including physical burdens. The occurrence of lumbar pain among healthcare workers can impair the functioning of the healthcare system as it can present a barrier to effective patient care, lead to the loss of working days, and impose an additional financial burden [5].

Moreover, it is quite possible that health professionals are exposed to increased physical and psychosocial stress due to hospital work during the pandemic of the novel coronavirus (COVID-19), which, in turn, could have a negative impact on the severity of lumbar pain [6,7,8,9]. Stress levels, job satisfaction, coping mechanisms, and psychological wellbeing have all been listed among the factors contributing to lower back pain in workers [8,9]. The World Health Organization (WHO) declared the COVID-19 outbreak a pandemic on 11 May 2020 [10]. People around the world were seriously affected by this pandemic, and many lives were lost. The surge in COVID-19 cases led to a strain on healthcare systems, shortages in medical supplies, lack of hospital beds, and healthcare personnel. Healthcare workers faced vast challenges dealing with the high influx of patients, particularly during the initial phases of the pandemic when knowledge about the virus spread, prognosis and treatment were limited. The pandemic also highlighted inequalities in access to healthcare and raised mental health concerns due to isolation, increased anxiety, and grief. Health workers, the most severely affected professional group, have been reported to be disproportionately affected by COVID-19 fears, work stress, social isolation, and discrimination. Healthcare workers were also found to experience similar problems in previous pandemics [11]. During the global crisis caused by the COVID-19 pandemic, healthcare workers have been a group at the greatest risk of infection during their work. The physical and mental wellbeing and morale of healthcare professionals are necessary for the continuation of healthcare services, and the COVID-19 pandemic was found to be a threat to health, and the resilience of healthcare professionals was needed. To propose any tailored preventive measures and interventions at the workplace, studies conducted in our specific setting were needed.

Therefore, this research aimed to determine the impact of COVID-19 on the prevalence of lumbar pain in healthcare professionals in the Clinical Centre of Montenegro, as well as to identify factors associated with the occurrence of lumbar pain.

## 2. Materials and Methods

This research was conducted as a cross-sectional study in the period 1 July–31 October 2021 at the Clinical Centre of Montenegro (CCM) in Podgorica, Montenegro. Montenegro, a Southeastern European country, has a universal healthcare system funded through mandatory health insurance contributions. Healthcare services are organised through primary healthcare centres, hospitals, and specialised healthcare facilities. CCM is a healthcare institution encompassing a hospital with more than 750 beds, a medical scientific and research centre, and a teaching base. It is the only medical institution in Montenegro that provides health services at the tertiary level of healthcare and conducts consultative specialist and sub-specialist health services.

The criteria for inclusion included being a doctor or a nurse employed full-time in CCM. The criteria for exclusion from the study were the interruption of work for more than 1 year, greater psychophysical trauma unrelated to the professional environment occurring in the short-term period before the study, and disagreement with participation in the research. The sample size was based on the estimate of the self-reported one-year prevalence of lumbar back pain reported in the literature, ranging between 50% and 80% and the total number of those employed in the Clinical Centre of Montenegro (2850), with a relative accuracy of 5% and a confidence interval of 95% was found to be between 227 and 339. The sample size was intentionally exceeded to allow for subgroup analysis. During the study period, all COVID-19 wards were visited, and all doctors and nurses present there were included in the study. Together with this, doctors and nurses were recruited from non-COVID-19 wards (orthopaedics, surgery, dermatology) and asked to complete the survey. All health professionals fulfilling the inclusion criteria and present onwards during the days of data collection were asked to participate in the study and were handed out printed questionnaires by the principal investigator. Out of the 400 distributed questionnaires, 392 were fully completed (response rate 98.0%).

For the purpose of this study, the standardised Modified Nordic questionnaire for the analysis of musculoskeletal symptoms and the EQ-5D-5L—questionnaire for measuring the quality of life associated with health were used, together with a set of general questions about the demographic and occupational characteristics of the respondents.

There were 26 general questions. The first eight close-ended questions were related to socio-demographic characteristics: gender, age, marital status, number of children, lifestyle habits (use of cigarettes, alcohol, sedatives), occupation and educational level of the respondents. Seven close-ended questions related to data on the working unit, duration of working in health care (years), working shifts (only one 8 h shift, two (morning and afternoon 8 h shifts), 12 h shifts, morning + nights (24 h)) and overtime work (more than 40 h/week), and type of employment (fixed-term or permanent contract). In the next three open-ended questions, respondents were surveyed in terms of body mass, body height and physical activity (defined as sports, fitness or recreational activities that cause an increase in breathing or heart rate, lasting at least 10 min, continuously). Respondents were asked how often they engaged in physical activity and based on their responses, they were grouped into one of 4 levels—never, monthly, weekly, and daily physical activity. The remaining eight close-ended questions were related to working with COVID-19 patients on COVID-19 wards during the pandemic, the type and length of engagement, and the self-perceived impact of the pandemic (measured on a 1–5 Likert scale) on workload, the adequacy of provided personal protection during pandemics, available knowledge and information, and personal finances.

A modified Nordic questionnaire for the analysis of musculoskeletal symptoms (the basis of which consists of the Standardised Nordic Questionnaire (SNQ)) for the analysis of musculoskeletal symptoms was used to examine lumbar pain in employees. The questionnaire used in the survey consisted of 12 questions, which the respondent answered through the five degrees of the Likert scale, and answers of “no/yes”. The SNQ, developed in 1987, is a self-administered questionnaire that was developed to identify and measure musculoskeletal problems. The SNQ is reported to be a valid, reliable, and feasible tool that shows good concordance with the functional clinical evaluation [12]. The presence of lower back pain was identified based on the self-report of lumbar pain in the past year [13].

EQ-5D-5L is a standardised questionnaire which measures the self-reported quality of life. It is applicable to a wide range of health conditions and treatments. EQ-5D-5L provides a simple descriptive profile over five dimensions and a single index value for health status. The EQ-5D-5L system five dimensions are: mobility, self-care, usual activities, pain/discomfort, and anxiety/depression. Each dimension has 5 levels: no problems, slight problems, moderate problems, severe problems, and extreme problems. EQ VAS records the patient’s self-rated health on a vertical visual analogue scale. The score values ranged between 0 and 100, with 100 being the maximum value. The EQ-5D index value can have a maximum value of 1 that reflects full health and quality of life. Convergent validity is demonstrated by a correlation between EQ-5D-5L and the dimensions of WHO 5 (r = 0.43, *p* < 0.001), and the average test–re-test reliability using inter-class coefficients has a mean of 0.78 and 0.73 [14,15,16].

All data were processed in the IBM SPSS Statistics 22.0 (SPSS Inc., Chicago, IL, USA) software package. For the analysis, the following were used depending on the type and distribution of the variables—the *t*-test for normally distributed numeric variables, the Mann–Whitney test for ordinal and non-normally distributed numeric variables, and the Chi-square test for categorical variables. Multivariate logistic regression with lumbar pain as the dependent variable was used to determine the factors associated with lumbar pain. Statistical hypotheses were tested at a level of statistical significance (alpha level) of 0.05.

## 3. Results

The study included 392 subjects, with the majority of females 252 (64.3%) and an average age of 38.1 ± 11.0 years. The one-year self-reported prevalence of lower back pain was 68.1%. The average body mass index (BMI) was 24.6 ± 3.4 kg/m^2^. Participants with lumbar pain did not report engaging in additional physical activity (35.3%), i.e., they exercised rarely (31.2%), while in the group of subjects without lumbar pain, the largest number of participants reported being physically active once a day (30.6%) (Table 1).

Regarding the occupation of respondents, approximately a third were physicians (31.9%), while the remaining two-thirds were nurses/technicians (68.1%). The presence of lumbar pain was found in a significant number of subjects, given that 267 subjects confirmed their experience in terms of lumbar pain, which makes up more than two-thirds of the examined population (68.1%). Subjects with lumbar pain had more working experience (15.1 ± 10.7 years) and most commonly worked in shifts of 12 h (41.2%) compared to subjects without lumbar pain (Table 2).

COVID-19 engagement, up to any degree, was significantly more common in subjects with lumbar pain (67.8%) compared to subjects without lumbar pain (57.6%). Subjects with lumbar pain had a higher degree of workload and over-engagement (65.8% vs. 54.4%, *p* = 0.042) (Table 3).

A significantly higher degree of mobility problems (41.9% vs. 12% *p* = 0.001) was found in those reporting lumbar pain than in those who did not. Subjects with lumbar pain were also significantly more likely to have problems with washing or dressing (26.2% vs. 3.28% *p* = 0.001) and more often had problems performing their usual activities (36% vs. 11.2% *p* = 0.001). Lumbar pain was also accompanied by significantly more frequent reporting of anxiety and depression (31.8% vs. 19.2%). Subjects with lumbar pain had significantly lower scores on the VAS self-assessment of health status (79.0 ± 17.5 vs. 88.1 ± 14.9 *p* = 0.001), as well as significantly lower EQ-5D index values (0.92 ± 0.11 vs. 0.98 ± 0.07 *p* = 0.001) (Table 4).

In the model of multivariate logistic regression, with the presence of lumbar pain as a dependent variable, factors associated with lumbar pain were a higher degree of physical inactivity (OR = 1.24, *p* = 0.042), a higher degree of over-engagement load during the COVID-19 pandemic (OR = 1.49, *p* = 0.014), and the duration of engagement during the COVID-19 pandemic (up to a month OR = 4.5, *p* = 0.004 and 1–3 months OR = 3.54, *p* = 0.025) (Table 5). With every degree of physical inactivity, the odds of reporting lumbar pain increased by 24%, and for every level of a 5-point Likert scale on the question about over-engagement during the COVID-19 pandemic, the odds of reporting lumbar pain increased by 49%.

## 4. Discussion

Assessing the frequency of lumbar syndrome among healthcare workers is of great importance because this syndrome leads to the disruption of the functioning of employees, can affect the quality of services provided, and withdraw employees from the workplace for a shorter or longer period [17]. The present study examined the prevalence of lumbar syndrome in healthcare workers employed at the Clinical Centre of Montenegro during the COVID-19 pandemic, as well as to identify risk factors. To our knowledge, such a study has not been conducted among the employees of the Clinical Centre of Montenegro before.

The presence of lumbar pain was found in a significant number of subjects, given that 267 subjects confirmed their experience in terms of having lumbar pain, which makes up more than two-thirds of the respondents (68.1%). Similar data were found in studies by other authors, and in a study by Cheung and collaborators conducted in Hong Kong, the prevalence of lumbar pain among healthcare professionals was 66.6% [18]. In some countries, the prevalence among healthcare workers is over 70% (Sweden 77%, Taiwan 72%) [19,20]. Other authors cite a slightly lower prevalence, and in a study by Guan et al. conducted in China, the prevalence was 54.8%, but it still accounted for over half of the surveyed population, which speaks in favour of the high occurence of this disorder among healthcare workers [21].

Factors associated with reporting lumbar pain in subjects were several years of service, as well as working shifts of 12 h. Subjects with lumbar pain had a higher perceived degree of workload and over-engagement (65.8% vs. 54.4%). This was also one of the factors associated with the presence of lumbar pain, identified by the use of multivariate logistic regression, where respondents with a higher perceived degree of workload were almost 50% more likely to develop lumbar pain. This aligns with previous research highlighting the adverse effects of extended working shifts on healthcare professionals. Prolonged periods of physical exertion, coupled with limited rest intervals, may contribute to fatigue and strain on the musculoskeletal system, predisposing health-care proffesionals to conditions such as LBP. In addition, higher patient load may lead to heightened physical demands, compromising their ability to maintain optimal body mechanics during patient care activities. A study conducted in Poland also found back pain to be associated with longer working time [22], concluding that back pain associated with professional activities is the consequence of overload. Other studies also found a connection between increased workload and the occurrence of back pain in healthcare workers [20,23,24]. Kuijer et al. reviewed systematically the risk factors for work-related back pain [25], and their meta-analysis revealed that lumbosacral radiculopathy syndrome can be considered a work-related disease depending on the degree of exposure to the aforementioned activities. Another meta-analysis determined that the risk of lower back pain is associated with different levels of work-related exposure, such as weight and lifting frequency and that this should form the basis of health policies at the workplace [26]. Longer daily working hours and a large number of patients to care for per shift should be discouraged in order to prevent musculoskeletal problems in health professionals.

A recent systematic review and meta-analysis examining the effects of the COVID-19 pandemic on the impact of low back pain [27] found a significant increase in LBP prevalence and intensity during the COVID-19 pandemic, influenced by many factors. We observed that COVID-19 engagement, to any degree, was significantly more commonly reported in those who had lumbar pain (67.8%) compared to those without lumbar pain (57.6%). This was also identified in multivariate logistic regression as a factor associated with the onset of lumbar pain. Being engaged during the COVID-19 pandemic led to higher odds of developing lumbar pain compared to workers who were not engaged in COVID-19 departments. This calls for consideration of potential contributing factors, including psychosocial stressors associated with the COVID-19 pandemic, which may exacerbate lumbar pain, highlighting the complex interplay between physical and mental health during this health crisis [28,29]. A possible explanation for the higher prevalence of lumbar pain in health workers working with COVID-19 patients includes a higher workload in health professionals involved in COVID-19 wards, a higher degree of COVID-19 infection in this high-risk population, and higher stress levels or other factors.

In the present study, subjects with lumbar pain had significantly lower scores on the self-assessment of health status, as well as significantly lower values of the EQ-5D index. Detrimental effects of back pain on quality of life are well described in the literature [30,31]. Chronic back pain, in particular, is associated with physical, psychological, and social consequences that impair the quality of life [30]. Physically, back pain is associated with limitations in mobility, reduced functional capacity, and disrupted sleep patterns [32]. The present study also found that subjects with lumbar pain reported a significantly higher degree of mobility problems, problems washing or dressing themselves, and problems with performing usual activities. Psychologically, persistent discomfort can lead to anxiety, depression, and impaired cognitive function. We also observed that lumbar pain was accompanied by the significantly more frequent reporting of anxiety and depression.

In addition to higher workload and COVID-19 engagement, one of the factors associated with the presence of lumbar pain, identified by the use of multivariate logistic regression, was physical inactivity, where a higher degree of physical inactivity was associated with 24% higher odds of experiencing lumbar pain. A systematic review identified sedentary lifestyle as a considerable risk factor for lower back pain (OR = 1.24, 1.02–1.5) as well as prolonged sitting time (OR = 1.42, 1.09–1.85) and driving time (OR = 2.03, 1.22–3.36), but concluded that research on the correlation between sedentariness and high-intensity lower back pain are scarce and inconclusive [33]. Another review found that sedentary behaviour does not appear to increase the chances of developing a new episode of low back pain and that health lifestyle contributors seem to be more related to the amount and type of physical activity, but not the amount of sedentary time [34]. Physical activity is beneficial for the prevention of lower back pain, with a general exercise program that combines muscular strength, flexibility and aerobic fitness proving effective in the treatment of chronic lower back pain [35].

This study has some limitations that need to be mentioned. The cross-sectional design of this study limited our ability to establish causal relationships between identified factors and lumbar pain. The study focused exclusively on health workers employed in the Clinical Centre of Montenegro, and the findings may not be generalisable to health workers in other settings or regions. The data collected in this study rely on self-reported measures, which introduces the possibility of recall bias. Some parameters that may be related to low back pain not examined in this study, are other comorbid conditions, psychological factors such as stress, dissatisfaction, demotivation, and psychological overloads. Despite these limitations, this study provides valuable insights that can inform future research and evidence-based preventive measures in this specific population in our setting.

## 5. Conclusions

The findings of the present study can help inform healthcare professionals, policymakers, and management structures on the burden of lower back pain and its associated risk factors, guiding the development of targeted interventions. Based on these findings, a comprehensive, multidisciplinary program aimed at the prevention and early detection of lower back pain among medical professionals is needed in our setting.

## Figures and Tables

**Table 1:** Demographic data for those with and without the presence of lumbar pain.

Characteristics	Lower Back Pain N (%)	No Lower Back Pain N (%)	*p*-Value
Sex			
Male	90 (33.7)	50 (40.0)	
Female	177 (66.3)	75 (60.0)	0.226 *
Age	38.7 ± 11.3	36.7 ± 10.4	0.097 **
Marital status			
Single	100 (37.5)	65 (52.0)	
Married	139 (52.1)	52 (41.6)	
Divorced	28 (10.5)	8 (6.4)	0.021 *
Number of children			
0	118 (44.4)	67 (53.6)	
1	35 (13.2)	14 (11.2)	
2	70 (26.3)	24 (21.6)	
3	39 (14.7)	13 (10.4)	
4	4 (1.5)	4 (3.2)	0.126 ***
BMI	24.8 ± 3.4	24.3 ± 3.4	0.212 *
Physical activity			
Daily	61 (22.9)	38 (30.6)	
Weekly	28 (10.5)	24 (19.4)	
Monthly	83 (31.2)	27 (21.8)	
Never	94 (35.3)	35 (28.2)	0.019 *
Total	267 (68.1)	125 (31.9)	

* Chi-square test; ** *T*-test; *** Mann–Whitney U test.

**Table 2:** Factors influencing the occurrence of lumbar pain.

Characteristics	Lower Back Pain N (%)	No Lower Back Pain N (%)	*p*-Value *
Occupation			
Doctor	76 (28.9)	46 (38.3)	
Nurse	187 (71.1)	74 (61.7)	0.066 *
Work experience (years)	15.1 ± 10.7	12.3 ± 9.2	0.018 **
Shifts			
Only one	54 (20.2)	26 (20.8)	
Two (morning and afternoon)	38 (14.2)	13 (10.4)	
12 h shift	110 (41.2)	43 (34.4)	
Morning + nights (24 h)	65 (24.3)	43 (34.4)	0.164 *
Working overtime ***			
Yes	204 (76.7)	87 (69.6)	
No	62 (23.3)	38 (30.4)	0.134 *
Total	267 (68.1)	125 (31.9)	

* Chi-square test; ** Mann–Whitney U test; *** more than 40 h/week.

**Table 3:** COVID-19 engagement and impact on lumbar pain reporting.

Characteristics	Low Back Pain N (%)	No Low Back Pain N (%)	*p*-Value *
COVID-19 engagement			
No	86 (32.2)	53 (42.4)	
Yes	181 (67.8)	72 (57.6)	0.049 *
COVID-19 engagement duration			
Not engaged	86 (32.2)	53 (42.4)	
Up to a month	39 (14.6)	7 (5.6)	
Between 1 to 3 months	24 (9.0)	5 (4.0)	
Between 3 to 6 months	25 (9.4)	11 (8.8)	
Longer than 6 months	30 (11.2)	14 (13.6)	
Longer than 12 months	63 (23.6)	32 (25.6)	0.650 **
Quarantine during COVID-19			
Yes	145 (54.5)	59 (47.2)	
No	212 (45.5)	66 (52.8)	0.177 *
COVID-19 infection			
Yes	99 (37.1)	51 (40.8)	
No	168 (62.9)	74 (59.2)	0.480 *
During the pandemic I had the following:			
Increased workload and excessive engagement			
Completely disagree	16 (6.0)	13 (10.4)	
Disagree	75 (28.2)	44 (35.2)	
Agree	133 (50.0)	54 (40.8)	
Completely agree	42 (15.8)	17 (13.6)	0.042 **
Adequate personal protective equipment			
Completely disagree	10 (3.7)	4 (3.2)	
Disagree	60 (22.5)	24 (19.2)	
Agree	165 (61.8)	81 (64.8)	
Completely agree	32 (12.0)	16 (12.8)	0.463 **
Enough knowledge on COVID-19			
Completely disagree	15 (5.6)	11 (8.8)	
Disagree	64 (24.0)	23 (18.4)	
Agree	105 (39.3)	43 (34.4)	
Completely agree	83 (31.1)	48 (38.4)	0.335 **
The pandemic impacted my finances			
Completely disagree	68 (25.6)	51 (40.8)	
Disagree	72 (27.1)	29 (23.2)	
Agree	87 (32.7)	28 (22.4)	
Completely agree	39 (14.7)	17 (13.6)	0.011 **
Total	267 (68.1)	125 (31.9)	

* Chi-square test; ** Mann–Whitney U test.

**Table 4:** Health-related quality of life determined by EQ-5D-5L.

Dimensions	Lower Back Pain N (%)	No Lower Back Pain N (%)	*p*-Value
Mobility			
I have no problem in walking about	155 (58.1)	110 (88.0)	
I have slight problems in walking about	67 (25.1)	13 (10.4)	
I have moderate problems in walking about	27 (10.1)	1 (0.8)	
I have severe problems in walking about	34 (5.2)	1 (0.8)	
I am unable to walk about	4 (1.5)	0 (0.0)	0.001 *
Self-care			
I have no problem washing or dressing myself.	197 (73.8)	121 (96.8)	
I have slight problems washing or dressing myself	50 (18.7)	2 (1.6)	
I have moderate problems washing or dressing myself	18 (6.7)	2 (1.6)	
I have severe problems washing or dressing myself.	2 (0.7)	0 (0.0)	
I am unable to wash or dress myself.	0 (0.0)	0 (0.0)	0.001 *
Usual activities			
I have no problem doing my usual activities.	171 (64.0)	111 (88.8)	
I have slight problems doing my usual activities	70 (26.2)	12 (9.6)	
I have moderate problems doing my usual activities	26 (9.7)	0 (0.0)	
I have severe problems doing my usual activities	0 (0.0)	2 (1.6)	
I am unable to do my usual activities.	0 (0.0)	0 (0.0)	0.001 *
Pain/discomfort			
I have no pain or discomfort	112 (41.9)	101 (80.8)	
I have slight pain or discomfort	83 (31.1)	20 (16.0)	
I have moderate pain or discomfort	64 (24.0)	2 (1.6)	
I have severe pain or discomfort	8 (3.0)	2 (1.6)	
I have extreme pain or discomfort	0 (0.0)	0 (0.0)	0.001 *
Anxiety and depression			
I am not anxious or depressed	182 (68.2)	101 (80.8)	
I am slightly anxious or depressed	66 (24.7)	17 (13.6)	
I am moderately anxious or depressed	14 (5.2)	6 (4.8)	
I am severely anxious or depressed	5 (1.9)	1 (0.8)	
I am extremely anxious or depressed	0 (0.0)	0 (0.0)	0.012 *
EQ VAS score (mean ± SD)	79.0 ± 17.5	88.1 ± 14.9	0.001 *
EQ index (mean ± SD)	0.92 ± 0.11	0.98 ± 0.07	0.001 *
Total	267 (68.1)	125 (31.9)	

* Mann–Whitney U test.

**Table 5:** Multivariate logistic regression model with lumbar pain as a dependent variable.

Characteristics	OR (95% CI)
Age	0.98 (0.94–1.03)
Marital status	
Unmarried	1.0 ref.cat.
Married	2.57 (0.84–7.85)
Divorced/widowed	1.85 (0.67–5.14)
Occupation (nurse vs. doctor)	0.95 (0.36–2.49)
Work experience (years)	1.04 (0.98–1.09
Working time	
Only the morning shift	1.0 ref.cat.
Two shifts (morning and afternoon)	2.60 (0.99–6.78)
12 h long shifts	1.29 (0.65–2.57)
Morning shift and on-call (24 h)	0.74 (0.27–2.02)
Degree of physical inactivity	1.24 (1.01–1.53)
COVID-19	
The degree of over-engagement	1.49 (1.09–2.05)
COVID-19 duration of engagement	
No engagement	1.0 ref.cat.
Up to a month	4.05 (1.57–10.49)
1–3 months	3.54 (1.17–10.73)
3–6 months	2.07 (0.86–4.97)
6–12 months	1.20 (0.53–2.71)
More than 12 months	1.37 (0.69–2.71)

## Data Availability

Data are available from the main author based upon individual request.
